# 1582. Dolutegravir/Rilpivirine STR Switch Study in Persons with HIV-1 and Chronic Kidney Disease: 48-week Assessment of Viral Suppression, Treatment Adherence and Quality of Life

**DOI:** 10.1093/ofid/ofad500.1417

**Published:** 2023-11-27

**Authors:** Helena Kwakwa, Tawanaha Stokes, Millen Gebreselassie, Lisa Maslankowski, Esther Chernak

**Affiliations:** Philadelphia Department of Public Health, Philadelphia, Pennsylvania; Philadelphia Department of Public Health, Philadelphia, Pennsylvania; Philadelphia Department of Public Health, Philadelphia, Pennsylvania; Philadelphia Department of Public Health, Philadelphia, Pennsylvania; Philadelphia Department of Public Health, Philadelphia, Pennsylvania

## Abstract

**Background:**

Single tablet regimens (STRs) are widely utilized in HIV treatment in the Western world largely because they improve convenience of oral regimens and eliminate differential adherence. US Blacks are disproportionately impacted by HIV, and are also disproportionately affected by chronic kidney disease (CKD) for a variety of reasons, especially in their later years. For PWH and CKD, STR options are limited due to pharmacokinetic considerations. We evaluate the impact on viral suppression, adherence, treatment satisfaction and quality of life (QOL) of switching to DTG/RPV STR for a largely Black aging US cohort with HIV and CKD.

**Methods:**

In this 48-week open label single arm switch study conducted in a community health center setting, adults with HIV, a GFR< 60 mL/min/1.73m^2^, an HIV RNA < 50 copies/mL and no known prior resistance to DTG or RPV were switched to DTG/RPV STR. HIV RNA and CD4 were monitored per standard national clinical guidance following switch. Standardized tools for medication adherence, treatment satisfaction and QOL were administered at baseline, 24 weeks and 48 weeks. We present final 48-week data.

**Results:**

Between August 2019 and December 2021, 35 participants were enrolled. One dropped out after 19 weeks and one after 22 weeks due to drug-drug interaction concerns following renal transplantation. 94% of participants were Black, 57% were female and 43% were 65 years and older. At baseline, 20% had a GFR < 30 mL/min/1.73m^2^ per race-based calculation (36% per race-free calculation). At 48 weeks, 97% of participants maintained viral suppression with an HIV RNA < 50 copies/mL in a per protocol analysis (91% in an intent-to-treat analysis). One participant with an HIV RNA of 62 copies/mL at 48 weeks subsequently re-suppressed on DTG/RPV. 69% reported an improvement in adherence, 77% noted an improvement in treatment satisfaction and 66% reported an improvement in QOL. The only adverse event reported in >5% of participants was nausea (5.7%). There were no serious adverse events.

**Conclusion:**

For this diverse aging cohort with HIV and CKD in a real-life community health center setting, DTG/RPV STR was efficacious, well-tolerated and associated with improvements in adherence, treatment satisfaction and QOL, sustained over 48 weeks. DTG/RPV is a feasible oral ARV option for PWH and CKD.

**Disclosures:**

**Helena Kwakwa, MD, MPH**, ViiV Healthcare: Grant/Research Support

